# SUMO-SIM Interactions Regulate the Activity of RGSZ2 Proteins

**DOI:** 10.1371/journal.pone.0028557

**Published:** 2011-12-06

**Authors:** Javier Garzón, María Rodríguez-Muñoz, Ana Vicente-Sánchez, María Ángeles García-López, Ricardo Martínez-Murillo, Thierry Fischer, Pilar Sánchez-Blázquez

**Affiliations:** 1 Cajal Institute, CSIC, Madrid, Spain; 2 CIBER of Mental Health, ISCIII, Madrid, Spain; 3 Department of Immunology and Oncology, National Centre of Biotechnology, CSIC, Madrid, Spain; Universidade Federal do Rio de Janeiro, Brazil

## Abstract

The *RGSZ2* gene, a regulator of G protein signaling, has been implicated in cognition, Alzheimer's disease, panic disorder, schizophrenia and several human cancers. This 210 amino acid protein is a GTPase accelerating protein (GAP) on Gαi/o/z subunits, binds to the N terminal of neural nitric oxide synthase (nNOS) negatively regulating the production of nitric oxide, and binds to the histidine triad nucleotide-binding protein 1 at the C terminus of different G protein-coupled receptors (GPCRs). We now describe a novel regulatory mechanism of RGS GAP function through the covalent incorporation of Small Ubiquitin-like MOdifiers (SUMO) into RGSZ2 RGS box (RH) and the SUMO non covalent binding with SUMO-interacting motifs (SIM): one upstream of the RH and a second within this region. The covalent attachment of SUMO does not affect RGSZ2 binding to GPCR-activated GαGTP subunits but abolishes its GAP activity. By contrast, non-covalent binding of SUMO with RH SIM impedes RGSZ2 from interacting with GαGTP subunits. Binding of SUMO to the RGSZ2 SIM that lies outside the RH does not affect GαGTP binding or GAP activity, but it could lead to regulatory interactions with sumoylated proteins. Thus, sumoylation and SUMO-SIM interactions constitute a new regulatory mechanism of RGS GAP function and therefore of GPCR cell signaling as well.

## Introduction

The regulator of G protein signaling (RGS)17 protein, also named RGSZ2, was initially described as a Gαo subunit-interacting protein [1], and subsequently, it was characterized as a GTPase accelerating protein (GAP) of several classes of Gα subunits, primarily Gi, Go, Gz, and Gq [2]. While no RGS protein displays avidity for the inactive GαGDP form, most RGS subfamilies exhibit weaker affinity for the GPCR-activated GαGTP form than for the GTP hydrolysis transition state, where GαGTPase initiates the conversion of GTP to GDP, [3], [4]. The RGS-Rz subfamily differs from other RGS proteins in that its members, RGS17, RGS19(GAIP) and RGS20(Z1), exhibit comparable avidities for both GαGTP and the transition state forms [5]. Binding of the GPCR-activated GαGTP subunit to its effectors generates this transition state and thus, the subunit is allowed to reach and regulate the effector before the binding of RGS proteins promote its deactivation. Thus, this unique characteristic displayed by RGS-Rz proteins has led to the proposal that they might fulfill an effector role [5]. Indeed, in brain RGSZ2 behaves as an effector that binds the neural nitric oxide synthase (nNOS) and negatively regulates the production of nitric oxide (NO) that is induced by the Mu-opioid receptor (MOR) agonist morphine [6].

The members of the RGS-Rz subfamily display notable differences in their distribution. RGSZ1 is primarily expressed in the brain [7], [8], whereas GAIP is abundant in peripheral tissues with only weak expression in the brain [9] and RGSZ2 is found in various tissues, including the brain [2], [10]. Interest in the physiology of the RGSZ2 protein has increased in recent years, particularly with a view to understanding the mechanisms regulating its function to certain human cancers. The *RGSZ2* gene is potentially behind the familial lung and bladder cancer susceptibility locus on chromosome 6q23–25 [11], [12], and the RGSZ2 protein is over expressed in both human lung and prostate cancer [13], [14]. The RGSZ2 has also been implicated in human cognitive ability [15], and the genome wide association database relates this gene to Alzheimer's disease, cerebral aneurysm, narcolepsy, and panic disorder (https://gwas.lifesciencedb.jp/cgi-bin/gwasdb/gwas_gene.cgi?name=RGS17). Indeed, 6q25 is one of the most relevant schizophrenia-susceptibility locus on this chromosome [16], [17].

Although various RGSZ2 transcripts can be found in different areas of the human brain, only a single transcript has been detected in peripheral tissues [2]. Indeed, despite the numbers of variants found, just two proteins are generated, each sharing a common structure: a 210 residue RGSZ2 protein (NP_064342) and a 230 residue RGSZ2 protein with a 20 amino acid extension at the N terminus (NP_001155294). Below we shall consider the different domains and regions of the 210 aa core RGSZ2 structure. This RGS protein contains a cysteine rich domain (CRD) in its amino-terminus (residues 28–40) and the RGS box (RH domain; residues 80–190, comprised of 9 alpha helices). The protein also contains several putative casein kinase 2 and PKC phosphorylation sites, and a series of PDZ domain binding motifs (61–64 MESI, 75–78 ADEV, and 76–79 DEVL) [2], [6]. Moreover, as described for other RGS-Rz member, GAIP [18], [19], the RGSZ2 could also attach to the cell membrane through palmitoylation of the CRD. The RH domain of this protein binds activated GαGTP subunits and regulates signaling at GPCRs, acting as an effector antagonist [2], [10], [20]. RGSZ2 and RGSZ1 bind to the histidine triad nucleotide-binding protein 1 (HINT1) at the MOR C terminus [21], and they contribute to the cross-regulation of the MOR and the *N*-methyl-D-aspartate receptor (NMDAR) [22]. By binding to the N terminus PDZ region of nNOS, the PDZ binding motifs of RGSZ2 serve to regulate the production of NO and the release of zinc from intracellular stores, thereby contributing to the activation of the glutamate-regulated NMDAR [6], [23]. In analogy to the previously described for RGSZ1 and GAIP, the RGSZ2 also is thought to interact with proteins such as SCG-10, synapsin-1a, GIPN [24]–[26]. As such, the RGSZ2 protein plays essential roles in regulating cell signaling through both metabotropic and ionotropic NMDA receptors.

The regulation of RGS GAP activity by post-translational modifications such as phosphorylation of RH critical residues, and to a minor extent palmitoylation is a relatively common process [27]. However, little is known about the cellular regulation of the multifaceted RGSZ2 protein. The presence of the RGSZ2 protein in neuronal nuclei suggests a regulatory role for this protein in gene transcription [28] and also opens the possibility that a novel mechanism, incorporation of Small Ubiquitin-like MOdifiers (SUMO), regulates its function. Sumoylation of proteins is a reversible modification that mechanistically resembles ubiquitination and that alters the activity of a broad range of (primarily nuclear) proteins [29]. Therefore, we considered it interesting to analyze the capacity of RGSZ2 to incorporate SUMO and how this covalent attachment would influence its GAP activity on GPCR-activated GαGTP subunits. Our results indicate that RGSZ2 co-localizes with GPCRs in the cell membrane where it can regulate the strength of the signals transduced by these receptors. Moreover, RGSZ2 GAP activity is finely regulated through the covalent attachment of SUMO in its RH domain, and by SUMO-interacting motifs (SIMs) that non-covalently bind SUMO.

## Results

### RGSZ2 contains a single SUMO attachment site and two SUMO-interacting motifs

**Figure 1:**
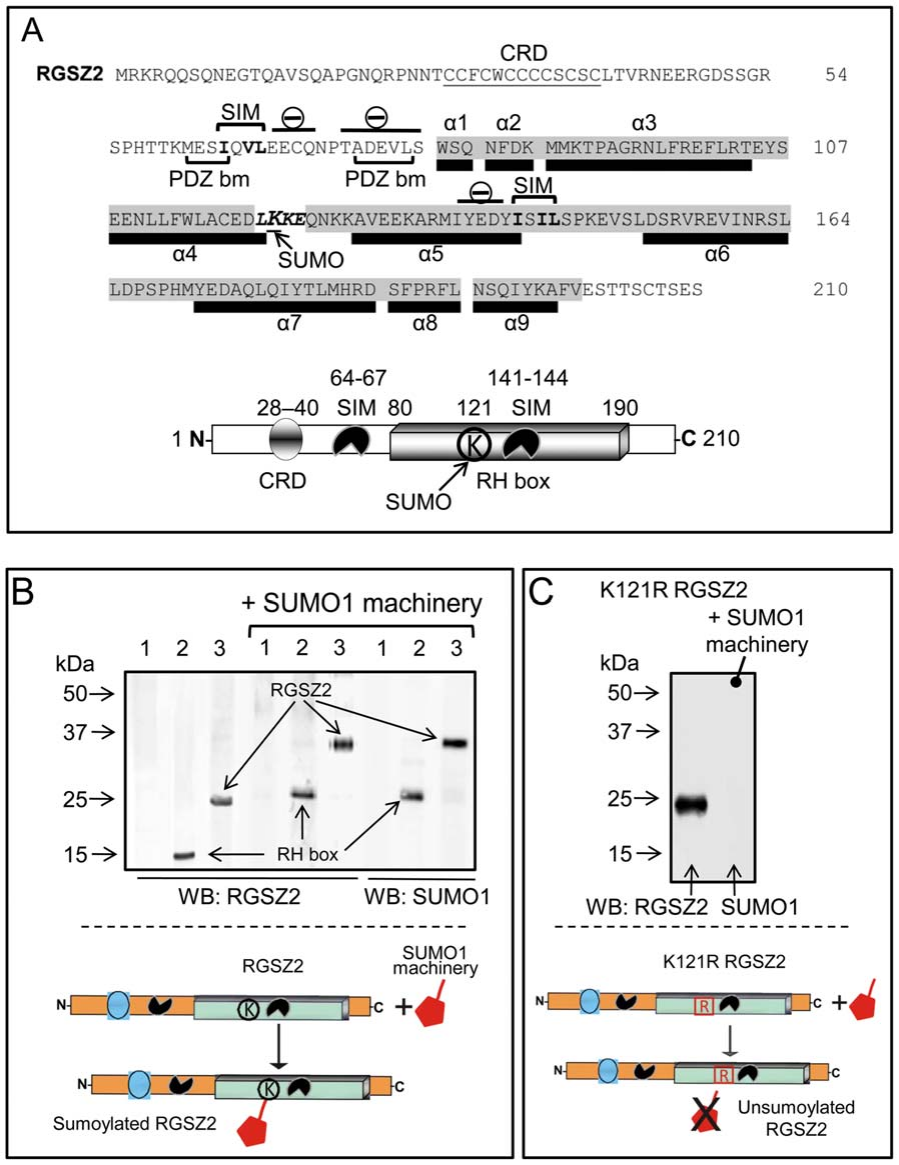
RGSZ2 contains a SUMO-attachment site and SUMO-interacting motifs. *A*, Protein domains of the murine RGSZ2 protein (NP_064342). The cysteine- rich domain (CRD) is underlined, the RH domain (RGS box) is shaded in gray and the α-helical residues of the secondary structures are indicated. The Small Ubiquitin-like Modifier (SUMO) consensus motif is in bold-italics, with lysine121 enlarged-underlined, SUMO-Interacting Motifs (SIMs) are in bold, indicating the clusters of negative amino acids surrounding the SIMs. The PDZ domain binding motifs (PDZ bm) are also indicated. Below, a diagram of the RGSZ2 protein indicates the position of the cysteine-rich domain, RH region, SUMO covalent modification K121 (LKKE), non-covalent SIM IQVL (64–67) and ISIL (141–144). *B*. The RGSZ2 and the RH region were cloned into E. coli BL21 (DE3) with or without the SUMO machinery [34]. GST-free RGSZ2 proteins (TEV cleavage) were resolved by SDS-PAGE, and probed with anti-RGSZ2 and anti-SUMO1 antibodies. Lane 1: control (no RGSZ2 vector); lane 2: RH region; lane 3: whole RGSZ2 sequence. *C*. The K121R RGSZ2 mutant induced along with the SUMO machinery was not sumoylated. Assays were repeated at least twice and produced comparable results. Representative experiments are shown.

The RGSZ2 protein contains 210 residues with a cysteine-rich domain in its amino-terminus (residues 28 to 40; 9 of 13 total residues are cysteine), a series of PDZ binding motifs (residues 61–64 MESI, 75–78 ADEV, and 76–79 DEVL) which binds to the nNOS PDZ domain [6], and the typical nine alpha helices of the RH domain (residues 80 to 190) (Fig. 1A). We had previously observed forms of the 24 kDa RGSZ2 protein that could be sumoylated in brain synaptosomes [6], [28]. Therefore, we considered of interest to address the impact of sumoylation on the RGSZ2 GAP activity. The RGSZ2 sequence predicts that, in addition to a sumoylation consensus site at the end of the α4 RH box (K121 in L*K*KE: Fig. 1A, http://www.abgent.com/doc/sumoplot), RGSZ2 contains two putative SUMO-interacting motifs (SIMs): one before the RH, IQVL (64–67), and the other at the end of α5 RH, ISIL (141–144). A typical SIM consists of a hydrophobic box of four aliphatic residues with the consensus sequence Val/Ile-X-Val/Ile/Leu-Val/Ile/Leu (V/I–X–V/I/L–V/I/L), juxtaposed to a negatively charged cluster. This structure allows the hydrophobic residues in SUMO, the β-grasp fold, and the neighboring positive charge to interact with the SIM [30]–[33].

To confirm that the predicted RGSZ2 sumoylation site was functional, we reconstructed the sumoylation pathway in *Escherichia coli* to obtain recombinant sumoylated proteins. The machinery was composed of human SUMO1, human Aos1, human Uba2, murine Ubc [34], as well as the target proteins RGSZ2 or its RH region. The SUMO1/2/3 proteins range from 10 to 12 kDa, and only the SUMO2/3 isoforms form polymeric SUMO chains [35]. To avoid branching, we restricted our analysis to the incorporation of SUMO1. Then, GST-RGSZ2 and GST-RGS box were co-expressed with the SUMO1 machinery. Afterwards, they were cleaved by TEV protease and GST removed. The SDS-PAGE analysis revealed that RGSZ2 and its RH incorporated a SUMO1 molecule, as indicated by a molecular weight shift of approximately 11 kDa and their recognition by anti-SUMO1 antibodies (Fig. 1B). No incorporation of SUMO1 was detected in a K121R RGSZ2 mutant (Fig. 1C) -evidence that the sumoylation site found in the RGS box is unique. The anti-RGSZ2 antibodies used in this study were purified and characterized by affinity binding against their antigenic peptide sequence, followed by their binding to the recombinant RGSZ2 protein ([6]; and Fig. S1).

**Figure 2:**
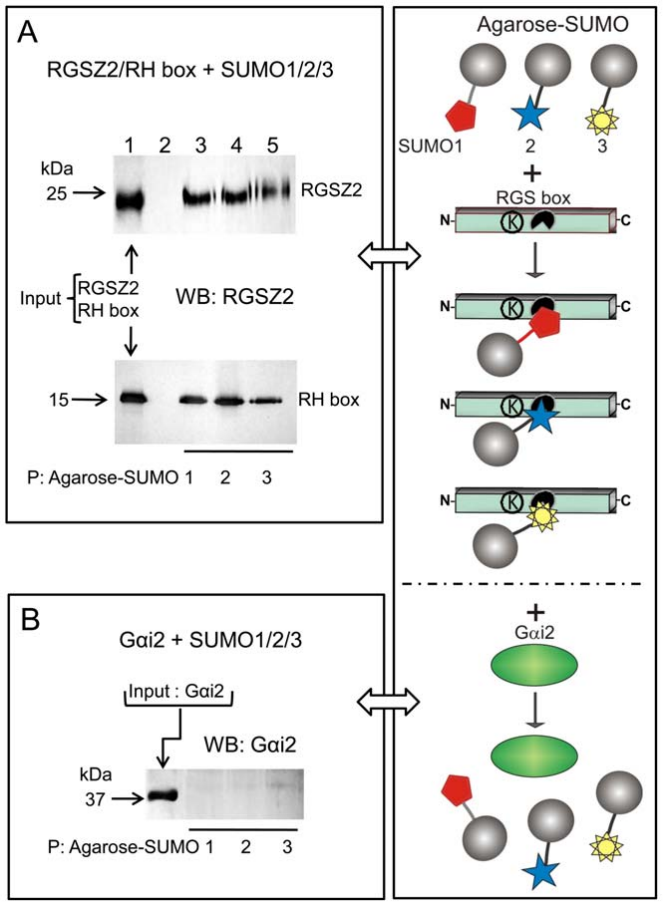
Sumo binds non covalently the recombinant RGSZ2 protein. *A*. Recombinant RGSZ2 and its RH region were incubated with SUMO1- (lane 3), SUMO2- (lane 4) and SUMO3-agarose (lane 5). SUMO-agarose conjugates captured RGSZ2 and its RH domain. Lane 1, RGSZ2/RH added. Lane 2, RGSZ2/RH in presence of agarose without SUMO. *B*. The Gαi2 subunit shows no binding to SUMO1/2/3-agarose.

**Figure 3:**
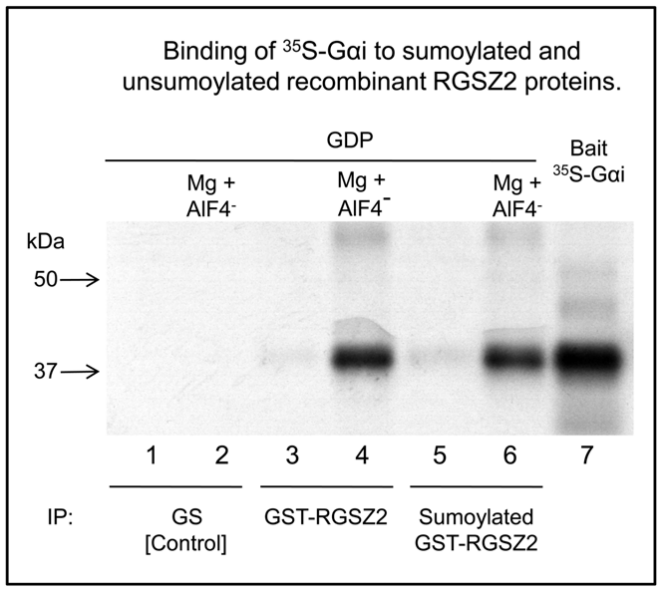
Influence of SUMO on Gαi subunit association with RGSZ2 proteins. Binding of ^35^S-Gαi to sumoylated and unsumoylated recombinant RGSZ2 proteins. In vitro-translated ^35^S-labeled GαiGDP subunits (10 µl) were incorporated into the samples, alone or with 2 mM MgCl2 and 30 µM AlF4- (30 µM AlF3 + 30 mM NaF). Samples in lanes 1 and 2 received glutathione sepharose (GS) beads; lanes 3 and 4, GST-RGSZ2 protein bound to GS beads; lanes 5 and 6, sumoylated GST-RGSZ2 attached to GS beads. Lanes 2, 4 and 6, 2 mM MgCl2 and 30 µM AlF4- were added to the incubation mixture. Lane 7 shows the Gαi, which was added to the samples analyzed in lanes 1 to 6. At the end of 2 h incubation, GS beads were precipitated and washed; RGSZ2-Gαi association was determined by autoradiography.

We tested the possibility of a sumoylated protein to interact with a SIM located on the RGSZ2 protein. Thus, agarose conjugated SUMO1, SUMO2 or SUMO3 exhibited non-covalent binding to RGSZ2 or its RH region (Fig. 2A). However, the Gαi2 subunit which contains two putative SIMs (VKLLL, residues 34 to 39; IILFL, residues 265 to 269) did not show stable binding to these SUMO variants (Fig. 2B). The RGSZ2 SIMs are juxtaposed to a negatively charged cluster and showed a notable avidity for its interaction with SUMO. In contrast, those on the Gαi2 protein lack the requested negative surrounding and the interaction was probably weak being disrupted during the precipitation procedure. From these assays we concluded that the RH, which is essential for binding to Gα subunits, contains at least one SIM able to bind to SUMO1, confirming computer-based predictions and the SUMO-SIM interactions in *trans*. The influence of the covalent incorporation of SUMO1 on the binding of the RGSZ2 RH to activated Gα subunits was evaluated by pull-down assays. It was observed that both RGSZ2 and its sumoylated form bound to Gα in the transition state (GDP + ALF4^-^). However, they bound poorly or not at all to GαGDP (Fig. 3). Thus, the RGS-attached SUMO1 did not prevent RGSZ2 from binding to the activated Gα subunits.

### The effects of SUMO and SUMO-SIM interactions on RGSZ2 GAP activity

**Figure 4:**
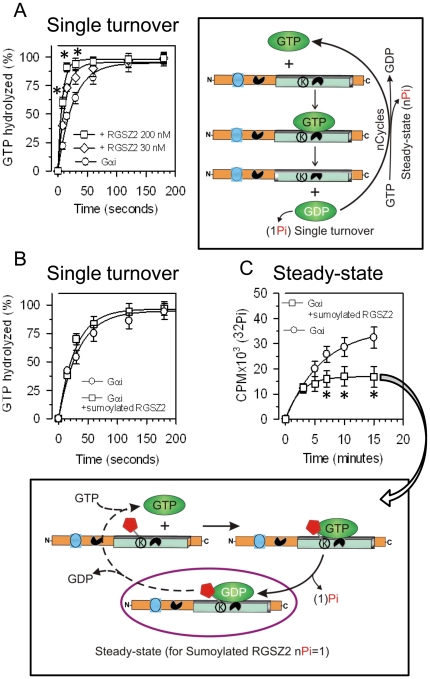
Effect of covalent sumoylation of RGS box on the GAP activity of RGSZ2 proteins. *A*. RGSZ2 GAP activity. In the single turnover assay, 100 nM Gαi was incubated with 30 or 200 nM of RGSZ2. The estimated *k*_cat_ (min^−1^) for Gαi when alone was 2.2; while in the presence of 30nM and 100nM RGSZ2 it was 4.4 (*k*_gap_ = 2.2 min^−1^) and 8.0 (*k*_gap_ = 5.8 min^−1^), respectively. The maximal release of Pi in these experimental conditions was about 1.1 pmol. *Significantly different from the value for Gαi alone; *P*<0.05. *B*. The effect of covalent attachment of SUMO1 on RGSZ2 GAP activity. Sumoylated RGSZ2 (200 nM) shows no GAP activity on 100 nM Gαi subunits in the single turnover assay, *k*_gap_ = 0.1 min^−1^. *C*. Sumoylated RGSZ2 blocks the liberation of Pi generated by Gαi GTPase in the steady-state. *Significantly different from the value for Gαi alone; *P*<0.05. For all assays, triplicate samples were collected at the intervals indicated and transferred to charcoal quenching solution. The values at time 0 were subtracted. Assays were repeated twice and the results were comparable.

Because covalently attached SUMO1 did not significantly alter the capacity of RGSZ2 to bind activated Gα subunits, we analyzed whether this modification affects its GAP activity by measuring the hydrolysis of GTP by Gαi in single-turnover assays with one cycle of Pi production. In the experimental conditions studied, the *k*_cat_ observed for recombinant Gαi was about 2.2 min^−1^, a value in the range described for recombinant Gαi/o proteins [36]. Recombinant RGSZ2 displayed concentration-dependent GAP activity on the Gαi-associated GTPase (Fig. 4A), and the sumoylated RGSZ2 did not significantly alter the basal GTPase activity (Fig. 4B). However, in steady-state assays in which n cycles of Pi production would be expected to occur, sumoylated RGSZ2 blocked Pi production (Fig. 4C). This result indicates that sumoylated RGSZ2 proteins bind to activated GαiGTP subunits but that they display weak or no GAP activity on the GαiGTPase. Moreover, the sumoylated RGSZ2 proteins delayed the release of GαiGDP subunits so that GDP could be exchanged for GTP and thus, the production of Pi diminished.

**Figure 5:**
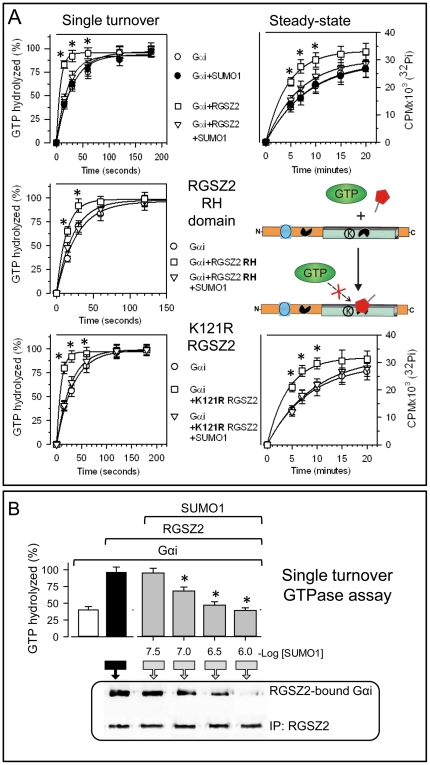
Non-covalent interaction of free SUMO1 abolishes RGSZ2 GAP activity. *A*. In single turnover and steady-state GTPase assays, the endogenous GTPase activity of 100 nM Gαi subunits was unaffected by 500 nM free SUMO1 in the medium, which nonetheless inhibited the GAP activity of 200 nM RGSZ2. The *k*_cat_ values (min^−1^) were: Gαi alone  = 2.1, Gα1 + SUMO1 = 2.0, Gαi + RGSZ2 = 7.9, Gαi + RGSZ2 + SUMO1 = 2,4. Free SUMO1 also impairs the GAP activity of the RGSZ2 RH domain. In single turnover assays of GTP hydrolysis by Gαi, the RH region shows GAP activity on Gαi subunits. This activity was blocked by the addition of SUMO1 to the medium. The *k*_cat_ values (min^−1^) were: Gαi alone  = 2.2, + RGSZ2 RH  = 4.0, + RGSZ2 RH and SUMO1  = 2.5. The RGSZ2 K121R mutant acts as a GAP on Gαi subunits, which is blocked by free SUMO1 in the medium. The *k*_cat_ values (min^−1^) were: Gαi  = 2.0, + K121R RGSZ2  = 6.9, + K121R RGSZ2 + SUMO1  = 2.1. *Significantly different from the value for Gαi alone; *P*<0.05. *B*. Concentration-dependent inhibition of RGSZ2 GAP activity by free SUMO1 -single turnover assay. The Gαi subunits were loaded with GTPγ^32^P alone or with RGSZ2 and increasing concentrations of SUMO1. The Pi release was determined 60 s after GTPase activation. *Significantly different from the value for Gα alone; *P*<0.05. Inset: At 60 s after initiation of the reaction, RGSZ2 was immunoprecipitated (IP; CT antibody) and the associated Gαi proteins were analyzed in Western blots probed with an anti-Gαi antibody.

To evaluate the influence that the non-covalent binding of sumoylated proteins to RGSZ2 SIMs could have on the GAP activity of RGSZ2, GTPase assays were performed in the presence of free SUMO1 (Fig. 5A). RGSZ2 and SUMO1 were used at equimolar concentrations. It was observed that SUMO1 interfered with RGSZ2 GAP activity without modifying the basal GTPase activity of Gαi. Similar results were obtained for free SUMO1 and the GAP activity of the RGSZ2 RH region or the mutated K121R RGSZ2 which does not incorporate covalently SUMO. However, in contrast to what was observed in the steady-state assay with sumoylated RGSZ2 protein, the RGSZ2 in the presence of free SUMO1 did not diminish Gαi-mediated Pi production. These data imply that free SUMO binds to the SIM inside the RGSZ2 RH and then reduces its GAP activity or simply prevents the access of the activated Gα subunit, leading to no detectable GAP activity.

**Figure 6:**
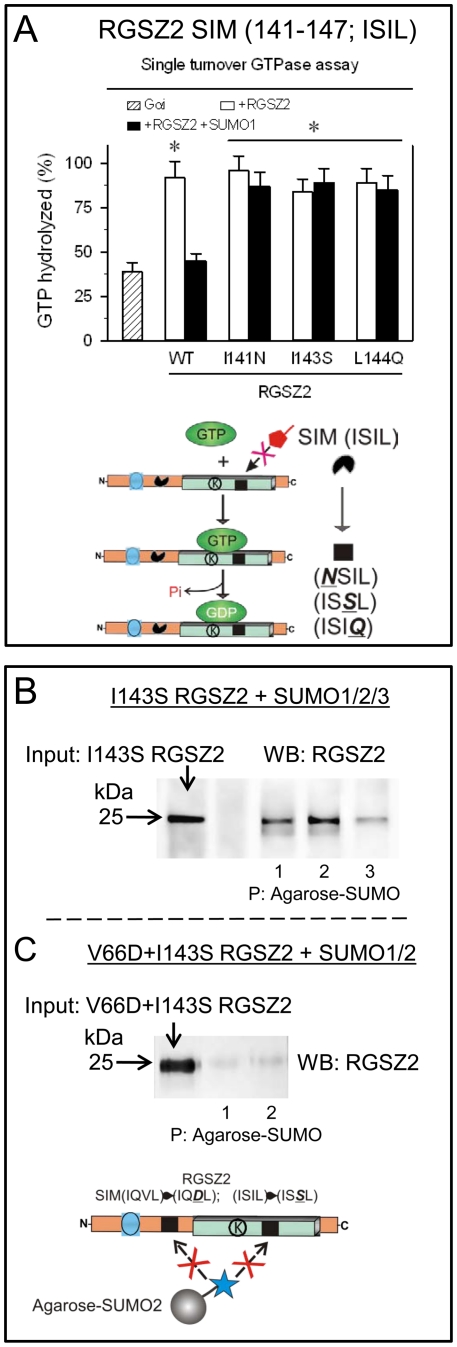
The RH domain SIM (141–144) binds SUMO1 and blocks RGSZ2 binding to Gα subunits. *A.* Disruption of the SIM (141–144) by mutation reverses free SUMO-mediated steric hindrance of RGSZ2-Gαi binding. The I141N, I143S and L144Q RGSZ2 mutants displayed GAP activity on Gαi, even in the presence of 1 µM free SUMO1. *Significantly different from the value for Gα alone; *P*<0.05. *B*. SUMO proteins bind the I143S RGSZ2 mutant but not the double V66D+I143S mutated RGSZ2 (*C*), indicating the presence of a second SIM upstream of the RGS box (see Fig. 1A).

These possibilities were evaluated by analyzing the influence of free SUMO1 on RGSZ2 GAP activity and Gα binding to the RH region. In single turnover assays, increasing concentrations of SUMO1 decreased the extent of RGSZ2-Gαi2 association (Fig. 5B). Thus, the blocking effect of free SUMO1 on RGSZ2 GAP activity correlated with a reduction in its avidity for the Gα subunit. The involvement of SIM (residues 141 to 144) in this antagonism was demonstrated by testing the GAP activity of RGSZ2 mutants in the presence of free SUMO1. Although wild-type RGSZ2 increased GTPase activity, this increase was prevented by SUMO1. However, the I141N, I143S and L144Q RGSZ2 missense mutants all displayed increased GTPase activity independently of the presence of free SUMO1 (Fig. 6A). We noted that the I143S RGSZ2 mutant continued to co-precipitate with SUMO proteins, which indicated the presence of at least one other SIM in the RGSZ2 molecule (Fig. 6B). By contrast, the V66D+I143S RGSZ2 double mutant did not associate with SUMO (Fig. 6C). Thus, RGSZ2 appears to have two SIMs, one upstream of the RH and another within the RH region (Fig. 1A).

### The effect of SUMO-SIM interactions on the cellular distribution and activity of endogenous RGSZ2

**Figure 7:**
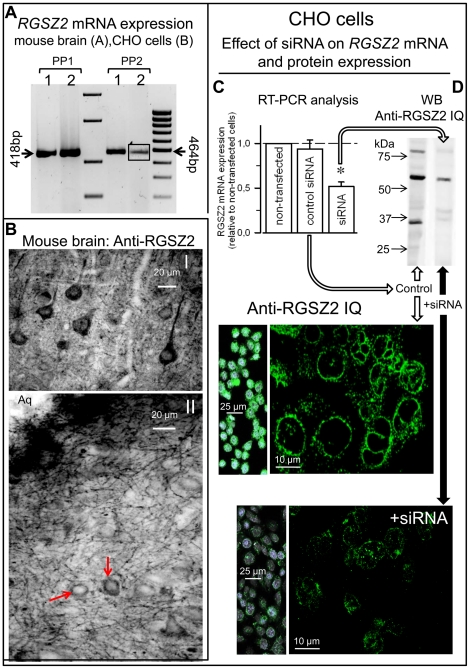
RGSZ2 mRNA and protein expression in mouse brain and CHO cells. *A*. Identification of RGSZ2 mRNA in mouse brain and CHO cells. One hundred nanograms of total RNA isolated from (1) mouse brain and (2) CHO cells were used for RT-PCR analysis. Arrows indicate the bp PCR product of RGSZ2 obtained using alternative primers pair PP1 & PP2, see Methods. DNA Ladders: 1550, 850, 400, 200; 1031, 900, 800, 700, 600, 500, 400, 300, 200 bp. The CHO 464 bp product (2) has been intensified. *B*. Light micrographs taken from coronal sections through the cerebral cortex (I) and PAG (II) illustrating the localization of the RGSZ2 immunoreactivity (IQ antibody). I, shows labeled neurons pyramidal in shape in layer V of the cerebral cortex. II, represents strong immunostaining labeling cells (arrows) and processes in the PAG. Aq: midbrain aqueduct. *C*. Reduction of *RGSZ2* mRNA expression. Confluent CHO cells (80–90%) were transfected with control siRNA or siRNA. Total RNA was extracted, retrotranscripted to cDNA and mRNA expression of *RGSZ2* gene analyzed by real-time PCR. *GAPDH* gene expression was used as internal standard. Bars are the mean + SEM of *RGSZ2* mRNA expression as percentage of control cells without being transfected. *Significantly different from the control siRNA, *P*<0.05. *D*. Reduction of *RGSZ2* mRNA expression brings about decreases in the levels of the encoded protein (IQ antibody). For western blot assays CHO cells were solubilized in Laemmli buffer with reducing agents. Immunocytochemistry of CHO endogenous RGSZ2. Nuclei stained with DAPI in blue. The diminishing effect of siRNA on CHO cells RGSZ2 expression was analyzed 48 h after transfection.

We analyzed whether the cellular distribution of RGSZ2 is consistent with its proposed role in regulating GPCR signaling. RGSZ2 mRNA was detected in the mouse brain and in CHO cells (Fig. 7A). The RGSZ2 was found in dense puncta along the plasma membrane (Fig. 7B & 7D), pattern that is compatible with RGSZ2 regulating GPCR function. The RGSZ2 protein is a 24 kDa protein but when resolved by SDS-PAGE, the endogenous protein is typically detected as a ladder with a series of higher molecular weight isoforms [10], [37]. As observed in the CNS, the RGSZ2 protein from CHO cells also resolves as a ladder and the 24 kDa RGSZ2 can barely be detected. Significantly, all these bands were sensitive to specific siRNA's when analyzed by SDS-PAGE gel chromatography, indicating they are related to RGSZ2 (Fig. 7C & 7D).

**Figure 8:**
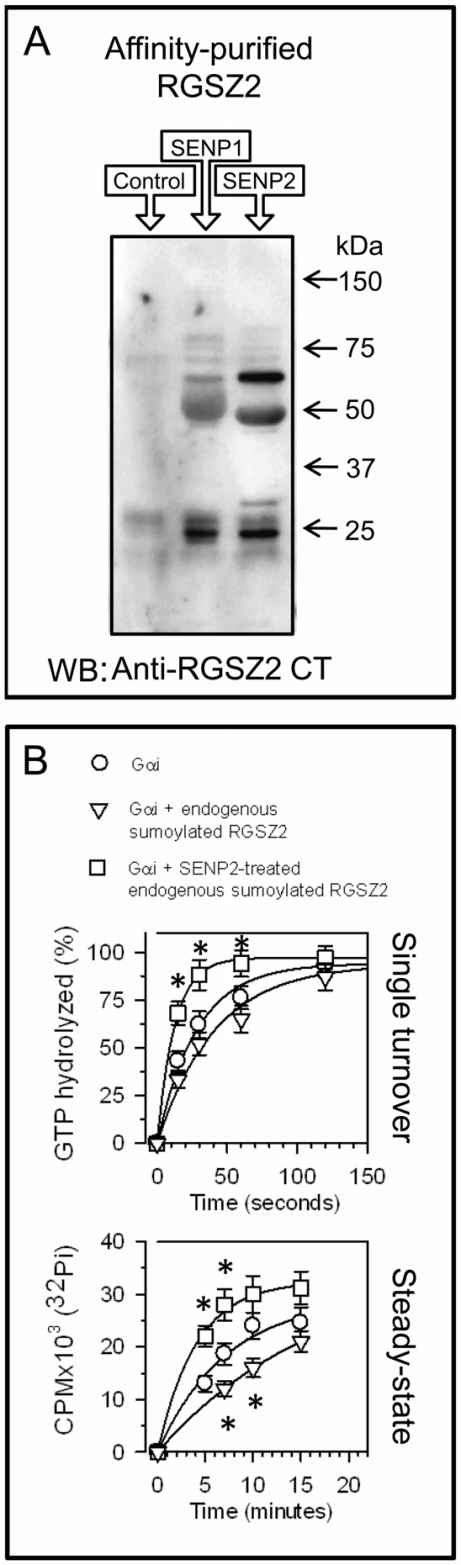
RGSZ2 GAP activity after SUMO removal. *A*. Effect of SUMO removal by SENP1 or SENP2 on neural RGSZ2 protein. RGSZ2 from SDS-octylthioglucoside solubilized synaptosomal membranes was retained by the NHS-agarose-coupled RGSZ2 antibody IQ. Affinity-purified RGSZ2 proteins were incubated alone (lane 1) or with proteases that remove SUMO (lanes 2 & 3). SENP1 preferentially removes SUMO1 whereas SENP2 removes SUMO1, SUMO2 and SUMO3. The samples were then solubilized in Laemmli buffer without reducing agents. In its absence and without SENP treatment, RGSZ2 heterocomplexes hardly enter the gel (lane 1). The anti-RGSZ2 CT was used to detect those signals. *B*. Sumoylated neural RGSZ2 protein lacks GAP activity but this is restored by SENP2 treatment. The observed *k*_cat_ (min^−1^) were: Gαi alone  = 2.1; in the presence of endogenous RGSZ2  = 1.8; and of SENP2-treated endogenous RGSZ2  = s.7. Data are the mean ± S.E.M. of triplicate samples collected at the intervals indicated. *Significantly different from the value for Gαi alone; P<0.05.

Thus, the covalent attachment of SUMO to the neural RGSZ2 protein seems to account for the different sizes of this protein observed in SDS-PAGE. Considering that sumoylation alters the activity of a broad range of proteins [29], we analyzed the influence of this modification on the regulation of neural RGSZ2 on activated GαGTP subunits. Accordingly, the neural RGSZ2 protein was enriched by affinity chromatography using agarose-coupled antibodies purified against peptide sequences from this RGS. In the absence of reducing agents, the neural RGSZ2 protein was hardly evident when the proteins recovered were resolved by SDS-PAGE (Fig. 8A, control lane). The SENP family of proteases produces some degree of protein de-sumoylation [38], and when the material pulled down by RGSZ2 antibodies was exposed to the action of the SENP1 and SENP2 proteases, RGSZ2 isoforms became visible in the gels, including the 24 kDa form. The presence of lower molecular weight bands after SENP treatment confirmed that RGSZ2 is mostly present in cells as a heavily sumoylated protein containing strings of SUMO2/3 [28] attached to the K121 site in the RH domain.

The effect of sumoylation on neural RGSZ2 GAP activity was addressed by measuring its influence on the rate of [γ-^32^P]GTP hydrolysis at Gα subunits (Fig. 8B). In single turnover assays Gαi hydrolysis of [γ-^32^P]GTP followed an exponential time-course and while the addition of sumoylated neural RGSZ2 did not affect the rate of Gαi hydrolysis, the treatment of this material with the SENP2 protease significantly accelerated Pi production resulting from GTP hydrolysis. Thus, increased GAP activity was linked to reductions in the amount of sumoylated RGSZ2. Indeed, in a steady-state GTPase assay in which GαGTPase activity depended not only on GTP hydrolysis but also on GDP/GTP exchange, sumoylated neural RGSZ2 decreased the Gα-mediated production of Pi in accordance with the results obtained for sumoylated recombinant RGSZ2 (see Fig. 4C). SENP2 treatment reversed this effect and increased the rate of GTP hydrolysis. These observations indicate that the sumoylated neural RGSZ2 protein can bind to GPCR-activated GαGTP subunits but it displays no GAP activity on them, rather it delays the release of GαiGDP subunits and impedes correct GTPase activity at the Gα subunits.

## Discussion

It is becoming frequently acknowledged that RGS proteins display a more complex role in cellular signaling than that expected for a GAP of activated GαGTP subunits. This is particularly notable for large RGS proteins in which the RH domain is just one of several protein interacting motifs. However, in RGSZ2 the RH domain represents half the total protein size but still, this RGS protein interacts with distinct proteins to regulate the activity of GPCRs (see Introduction). The present study indicates that the GAP activity and associated functions of RGSZ2 can be regulated by a novel mechanism, the covalent sumoylation of its RH domain, in concert with non-covalent interactions of SUMO-containing proteins at two SIMs: one within and the other outside of the RGSZ2 RH region.

The regulation of RGS RH GAP activity by post-translational modifications is a relatively common process. Phosphorylation of critical residues in the RH domain, and to a minor extent palmitoylation, can modify the GAP activity of these proteins [27]. Indeed, the GAP activity of the RGS-R7 subfamily is modulated dynamically by tumor necrosis factor-α which promotes the phosphorylation-dependent binding of 14-3-3 proteins [39]. Binding of 14-3-3 inhibits RGS-R7 GAP function and it is mediated by a conserved phosphoserine located in the Gα-interacting region of the RH domain [40]. Therefore, phosphorylation, palmitoylation, and now sumoylation can act as molecular switches to reduce or even abolish RH GAP activity. Such functional modifications may serve regulatory purposes and indeed, the non-covalent binding of SUMO to the RGSZ2 RH SIM located at residues 141–144 abrogates its interaction with GαGTP subunits. By preventing RGSZ2-GαGTP binding, this particular SUMO-SIM interaction enhances the signaling of the regulated GPCR to its effectors.

Notably, modified RH domains and proteins containing RH-like domains that can bind to GαGTP subunits but exhibit little or no GAP activity on them reduce the signaling capacity of GPCRs [41]. This is a process referred to as effector antagonism, and it is observed for GRK2 and GRK3, proteins that carry RH domains displaying little or no GAP activity but that sequester activated GαqGTP subunits, thereby attenuating the activation of PLCβ and reducing the signaling capacity of the corresponding GPCR [42]. Another example is the RH of Axin which binds to Gα12GTP subunits without affecting intrinsic GTP hydrolysis of Gα12, although it competes for binding to the RH domain of G12-interacting proteins, such as p115RhoGEF [43]. Accordingly, the persistent binding of the sumoylated RGSZ2 RH domain to effector active GαGTP subunits could be considered as effector antagonism as it delays their return to the control of the GPCR, reducing its signaling capacity [2], [10], [20]. In fact, morphine promotes the PKC-dependent transfer of MOR-regulated Gα subunits to RGSZ2 proteins, subsequently reducing the analgesic effects of opioids [10], [20]. Notwithstanding, RGSZ2 itself is as an effector of GPCR-activated GαGTP subunits. When the RGSZ2 RH domain remains bound to activated GαGTP subunits, this activity triggers the PKC-mediated separation of the RGSZ2-nNOS complex from the HINT1 protein [22], and the subsequent GαGTP.RGSZ2-mediated regulation of nNOS [6].

Sumoylation is widely considered as a nuclear or perinuclear phenomenon, as SUMO is covalently linked to many nuclear proteins, however, there is increasing evidence that plasma membrane proteins can also be modified in this manner [44], [45]. Examples of non-nuclear sumoylated proteins include Axin [46], the phosducin regulator of Gβγ signaling [47] and the RGS-Rz proteins that regulate Gα subunits [28]. These proteins may be subject to nucleocytoplasmic cycling facilitating their sumoylation in the nucleus, at nuclear pore complexes or even in the cytoplasm [48], [49], before they are transported to the cytosol and/or cell membrane. The RGSZ2 proteins probably undergo sumoylation in the nuclear fraction [28] prior to their transport to the neuronal membrane, where they through SUMO-SIM interactions influence GPCR signaling. In the SUMO-SIM interaction the SUMO protein folds into a complex structure that buries the hydrophobic SIM residues in a surface cleft, an interaction that is stabilized by hydrogen bonds between SIM residues and SUMO surface side chains [30]. The neighboring positive charge of SUMO interacts with the accessory negatively charged SIM cluster [30]–[33]. The quality of this ionic interaction is decisive in stabilizing the SUMO-SIM interaction, which may be almost irreversible, and it probably requires phosphorylation to disrupt the complex. Indeed, strong SUMO-SIM interactions have been observed through pull-down assays [50] that were absent when the putative SIM domains lacked the required accompanying negative sequence (*e.g.,* the Gαi2 subunit). As such, the reported affinity equilibrium constants (*KD*) of about 1–3 µM do not reflect a situation in which the strength of the SUMO-SIM interaction greatly reduces their spontaneous dissociation.

The presence of covalent SUMO modifications and of SIMs in the same molecule could lead to SUMO-SIM interactions assembling high-order protein complexes. This has been elegantly described for the promyelocytic leukemia protein (PML) that contains conjugated SUMO and SIM domains capable of promoting the formation of PML nuclear bodies [51]. The heavy sumoylation of RGSZ2 proteins has been confirmed using SENP proteases, which hydrolyze SUMO conjugated to target proteins, although these proteases only partially remove branched SUMO residues from proteins *in vitro* [38], [52], [53]. Both SUMO2 and SUMO3 also participate in neural RGSZ2 protein sumoylation [28] and these SUMO variants can form poly-SUMO chains that enhance the probability of interactions with SIM-containing proteins. Indeed, these variants could also participate in the recruitment of RGSZ2 proteins into protein complexes, as observed for SUMO-SIM interactions that regulate the association between critical nuclear proteins [54]. Accordingly, non-covalent binding of SUMO to RGSZ2 SIM 64–67 outside the RH should not affect Gα binding or RGSZ2 GAP activity but rather, these interactions could facilitate the role of RGSZ2 proteins as a scaffold, thereby influencing GPCR signaling [2], [10], [28].

In summary, SUMO-SIM interactions serve to localize and target proteins, as well as to modify protein function, as described here for the RGSZ2 protein. The covalent attachment of SUMO switches the activity of the RGSZ2 RH from that of a GAP of activated GαGTP subunits to that of an effector recognition site for GPCR-regulated Gα subunits. While the GAP and effector activities can be regulated through SUMO-SIM interactions at the RGSZ2 RH domain, those outside the RH preserve its GAP activity, probably contributing to the formation of RGSZ2 nucleated protein complexes. Thus, the influence of RGSZ2 proteins on GPCR signaling can be precisely and efficiently regulated through SUMO-SIM interactions. Alterations to this novel process may affect the contribution of RGSZ2 to the critical events in which it is involved.

## Materials and Methods

### Reagents

The phosphatase inhibitor cocktail, H89, polyoxyethylene10 lauryl ether (C12E10), phenylmethylsulphonyl fluoride (PMSF), leupeptin, aprotinin NP-40 and the protease inhibitor cocktail were obtained from Sigma. The Western blot Chemiluminescent HRP substrate was from GE Healthcare (RPN232), and Gαi were from Calbiochem. Small interfering RNAs (siRNA; sc-29528) against RGSZ2 (sc-61467), control siRNA (scRNA; sc-37007), fluorescein-conjugated control siRNA (sc-36869) were purchased from Santa Cruz Biotechnology, and SENP2 [368–549] fragment, GST-tagged (human, recombinant) was from Enzo Life Sciences (UW9765).

### Animals

Male albino CD-1 mice weighing 22–25 g were housed and used strictly in accordance with the European Community guidelines for the Care and Use of Laboratory Animals (Council Directive 86/609/EEC). The experimental protocols were reviewed and approved by the Committee for Animal Experimentation at the CSIC.

### Antibodies

Affinity-purified IgGs against the antigenic peptide: anti-RGSZ2 CT (C terminus amino acids 192–215), antibodies against peptide sequences in the Gα helical domain of Gαi2, have been described elsewhere [55], [56]. The anti-RGSZ2 W15 was purchased from Santa Cruz (sc-48286) and an anti-RGSZ2 IQ (internal sequence) was raised against the murine amino acid sequence 46–60 (GenScript, Piscataway, NJ). The anti-SUMO1 was obtained from Cell Signaling Technology. All primary antibodies were detected using the appropriate horseradish peroxidase-conjugated secondary antibodies.

### Preparation of synaptosomal membranes

The periaqueductal gray matter (PAG) and cortex from cohorts of 6-10 mice were homogenized (Polytron) in 25 mM Tris-HCl [pH 7.4], 0.32 M sucrose, H89, protease and phosphatase inhibitor cocktails. Homogenates were centrifuged (1000xg) to obtain post nuclear-supernatants (S1), which were processed to prepare synaptosomes. The synaptosomal pellet was resuspended in 50 mM Tris-HCl [pH 7.7] supplemented with 2mM PMSF, 2 µg/mL leupeptin and 0.5 µg/mL aprotinin. The membranes were aliquoted and frozen at −80°C.

### Preparation of endogenous RGSZ2 proteins by immunoaffinity chromatography

Synaptosomal membranes P2 were thawed and sonicated (two cycles of 5 s each) in a buffer containing 50 mM Tris-HCl [pH 7.7], 50 mM NaCl, 1% Nonidet P-40 (NP-40), a phosphatase and protease inhibitor cocktail and H89. The proteins were then heated for 10 min at 40°C in 40mM Tris-HCl, 2% SDS buffer, cooled to room temperature and filtered (0.22 µm). The SDS concentration was reduced by adding octylthioglucoside to a final percentage of 0.65% and the samples were incubated overnight with anti-RGSZ2 CT coupled to NSH-activated Sepharose 4 Fast Flow beds (GE Healthcare Bio-Sciences). After washing, the immune complexes were collected by an acid wash (100 mM Glycine-HCl [pH 3]), neutralized to pH 7.5 and the buffer was exchanged for a GTPase assay buffer. This procedure yielded RGSZ2 protein as verified by silver staining and Western blot analysis [28]. Incubation of the mSENP2 homologue that cleaves SUMO1, 2, and 3 conjugates, was used to remove SUMO from the immunoprecipitated RGSZ2.

### Expression of recombinant proteins in *Escherichia coli* and the sumoylation of RGSZ2 proteins

Murine full-length RGSZ2 (NM_019958) and its RGS box were cloned by PCR from PAG cDNA. Glutathione S-transferase (GST) fusion proteins were obtained by cloning mRGSZ2 and its RGS box into the pFN2A (GST) Flexi vector (Promega). The vector was introduced into *E. coli* BL21 (KRX, Promega, #L3002) and clones were selected on solid medium containing ampicillin. Bacterial lysates containing GST fusion proteins were immobilized on glutathione-Sepharose 4B columns (Amersham Biosciences), the GST was clipped off with ProTEV protease (Promega) and the GST-free proteins were concentrated. Finally, the TEV protease was removed by affinity chromatography.

Site directed mutagenesis was performed with Accuprime Pfx DNA Polymerase (Invitrogen) using pFN2AmRGSZ2 as the template. Lys 121 was mutated to Arg (K121R), Ile 141 to Asp (I141N), Ile 143 to Ser (I143S) and Leu 144 to Gln (L144Q) the amplified fragment was digested with NcoI and BamHI enzymes and cloned into pFN2A (GST) Flexi® Vector (Promega, Madison, WI, USA). To generate the I143S, V66D RGS17 double mutant PCR amplification was performed using as a template the plasmid pFN2ARGSZ2I143S. The sequences of the plasmids and the K121R, I141N, I143S, L144Q point mutations and I143S - V66D double mutations were confirmed by automated capillary sequencing.

For Sumoylation of recombinant RGSZ2, its RGS domain and K121R RGSZ2, the RGSZ2-containing vectors were electroporated into *E. coli* BL21 (DE3) alone or with a polycistronic pBADE12 vector encoding Aos1, Ubc9, Uba2 and pKRSumo1[34]. Transfected clones were selected on solid medium containing kanamycin, choramphenicol and ampicillin. Sumoylated proteins were resolved on 4–12% NUPAGE Bis-Tris gels (Invitrogen) and transferred to PVDF membranes that were probed with antibodies against SUMO1 or RGSZ2.

### SUMO and Gα subunit binding to RGSZ2 proteins

RGSZ2 (WT or RH domain) were incubated in 50 mM HEPES [pH 8.0], 1mM EDTA, 1 mM DTT, 0.05% C12E10 (for 2 h at room temperature) with SUMO1-, SUMO2- or SUMO3-agarose (BIOMOL). After washing, the beads were boiled in sample buffer, and the proteins were separated by SDS-PAGE and then transferred to PVDF membranes. The RGSZ2 bound to SUMO-agarose was determined by immunoblotting, detecting the anti-RGSZ2 with an HRP-conjugated anti-rabbit antibody. Chemiluminescence was recorded with a ChemiImager IS-5500 (Alpha Innotech, San Leandro, CA).

### GTPase assays

For single turnover GTP hydrolysis, 100 nM Gαi was loaded with 1 µM GTP plus 0.4 nM [γ-^32^P]GTP (6,000 Ci/mmol, Perkin Elmer) for 30 min in 50 mM HEPES [pH 8.0], 5 mM EDTA, 1 mM DTT, and 0.05% C12E10 at 30°C, after which the reaction mixture was shifted to 17°C. Reactions were initiated by adding a RGS mix consisting of 15 mM MgSO_4_, 150 µM GTP, 0.5 µM SUMO1 (BIOMOL) and/or 0.2 µM RGSZ2 protein (neural or recombinant), and the reactions were then quenched by adding charcoal, as described [24]. In blanks (without Gα subunits), 1–2% of total radioactivity added was evident as the background. Data were fitted to a single exponential curve (Sigmaplot v12, Systat Software, Inc. Germany) to obtain the GTP hydrolysis rate constant of the Gαi in the absence and in the presence of the potential modifier, e.g., RGSZ2 variants and SUMO1. RGS protein-dependent Gαi GTPase activity (*k*_gap_) was defined as [*k_cat_*(Gαi basal+RGS) - *k_cat_*(Gαi basal-RGS)] [57], [58].

Steady-state GTPase activity was measured as described elsewhere [59]. Briefly, heterotrimeric G proteins or Gα subunits plus SUMO1 and/or RGSZ2 proteins (neural or recombinant) were added to the reaction mixture containing 1 µM GTP plus 0.4 nM [γ-^32^P]GTP in 50 mM HEPES [pH 8.0], 1mM EDTA, 1 mM DTT, 15 mM MgSO_4_ and 0.05% C12E10, which was then incubated for 0–20 min at room temperature. The reaction was terminated by adding charcoal and the associated radioactivity was counted as above.

### RGSZ2 protein expression and mRNA

Deeply anaesthetized animals (Equithesin, Janssen Laboratories, 2.5 mL/kg intraperitoneally) were ventilated and perfused through the left ventricle with 0.9% saline followed by 250 mL of a fixative solution containing 4% paraformaldehyde in 0.1 M phosphate Buffer (PB), pH 7.4 After fixation and cryoprotection, serial 40 µm thick transverse frozen sections were cut with a 2800 Frigocut (Reichert-Jung) microtome and then processed. Immunohistochemistry for RGSZ2 was carried out in free-floating sections that were preincubated in 1% H_2_O_2_ in PBS for one hour to inactivate endogenous peroxidase. Sections were then treated with 3% normal goat serum, for 1 h at RT. After washing in PBS, the sections were incubated overnight at 4°C using the anti-RGSZ2 antibody IQ diluted (1∶500) in phosphate-buffered saline (PBS) containing 0.2% Triton X-100. After washing thoroughly in PBS, histological sections were incubated with biotinylated secondary goat anti-rabbit immunoglobulins diluted 1∶200 (Vector Laboratories, Burlingame, CA, USA) for 1 h at RT. After additional washes, sections were incubated with peroxidase-linked ABC (Vector Laboratories) for 90 min [60]. Peroxidase activity was developed as described [61]. Briefly, sections were preincubated in a histochemical medium that contained 0.06% 3,3′-diaminobenzidine tetra HCl (DAB, Sigma, St Louis, MO, USA) dissolved in PBS, for 10 min at RT, and then in the same solution containing 1 µL of 3% H_2_O_2_ per mL of DAB medium. The DAB reaction was interrupted at times chosen by inspection of trial sections (approximately 5 min). Control sections were routinely processed by either omitting the primary antibody or replacing it with an equivalent concentration of rabbit IgG. After the DAB reaction, histological sections were washed in PBS, mounted and dehydrated for light microscopy visualization. Histological sections were finally examined with a Zeiss Axiophot II microscope (Zeiss Iberica, Madrid, Spain) and images captured with a digital camera (DMC Ie, Polaroid, Cambridge, MA, USA).

CHO cells were grown in Dulbecco's modified Eagle's medium supplemented with 1 mM sodium Pyruvate, 2 mM L-glutamine, 100 U/mL streptomycin, 100 µg/mL penicillin and 10% (v/v) fetal bovine serum at 37°C in 95% air/5% CO_2_. Transfection of CHO cells with siRNA targeting mouse RGSZ2 and control siRNA was performed in LabTek 8-well chambers, with cells at 70–90% confluence, using siRNA transfection reagent following the manufacturer's instructions. The final concentration of the vector in the medium was 1 µg. The transfection efficiencies were estimated using a control siRNA fluorescein conjugate. The cells were evaluated by PCR and immunocytochemistry 48–72 h after transfection. For protein detection the cells were fixed with methanol and the RGSZ2 expression was analyzed by immunocytochemistry using the anti-RGSZ2 antibody (IQ 1∶200, 2h, room temperature) detected for 1h with an Alexa-conjugated goat anti-rabbit (1∶300; Molecular Probes, UK). After washing, cells were counterstained with DAPI and visualized on a Leica DMIII 6000 CS confocal fluorescence microscope equipped with a TCS SP5 scanning laser.

Total RNA was extracted from mouse brain, control cultures, siRNA transfected and siRNA control using the Illustra RNAspin Mini RNA isolation kit from GE Healthcare (Buckinghamshire, UK). Equal amounts of RNA were reverse transcribed using the First Strand Synthesis kit from Fermentas following the manufacturer instructions. Two different sets of primers were used, the sequences were the following, primers pair 1: GTTCGCGATCGCCTGGTCTCAGAATTTTGACAAGATG (forward); GATGGTTTAAACTTAGGATTCAGAAGTACAGCTGG (reverse); product length 418 bp; and primers pair 2: CTGAAGCCATGGACATGAGAAAACGG (forward); GCTGACCTCTTTCGGTGACTGTATAGAAATGT (reverse); product length 464 bp. Experiments were performed in Mastercycler (Eppendoorf) with 35 cycles as follows: 95°C for 20 s, 58°C for 20 s, and 68°C for 40 s after pre-incubation at 95°C for 2 min. PCR products were separated on 2.5% agarose gel with SYBR Safe™ DNA gel stain to verify the expected product sizes.

### Quantitative real-time polymerase chain reaction (PCR)

After reverse transcription (RT), the cDNA was diluted 1∶3 in nuclease-free water and used as template. RT-qPCR reactions were performed with SYBR green dye technique in a total volume of 20 µl using SYBR Green master mix in an ABI Prism 7500 Sequence Detector (Applied Biosystems) with conventional cycling parameters. Primer sequences designed using Primer Express (Applied Biosystems) were: CCCAACAATACCTGCTGCTTCT and GGACCTGGATGCTCTCCATTT. Glyceraldehyde 3-phosphate dehydrogenase (GADPH) was selected as control housekeeping gene. Melting curve analysis of each sample was performed after every run by defined heating up to 95°C to assess the presence of unspecific PCR products. For each biological replicate dual replicates were run. A minus RT control (RT-ve) was included in each assay run besides a negative water control reaction to assess for a possible contamination in the samples. All reactions were performed in triplicate and the quantities of target gene expression were normalized to the corresponding GAPDH expression in test samples and plotted.

### Statistical analysis

All data were expressed as the mean±SEM. Comparisons of the means between the experimental groups were performed with ANOVA followed by the Student-Newman-Keuls test. Differences with a *P* value of <0.05 were considered significant.

## Supporting Information

Figure S1 Supporting figure
(TIF)
