# Supplementary figures and images for: SUMO-SIM Interactions Regulate the Activity of RGSZ2 Proteins
Source: PLoS One. 2011 Dec 6;6(12):e28557. doi: 10.1371/journal.pone.0028557 (PMC3232247; doi:10.1371/journal.pone.0028557)

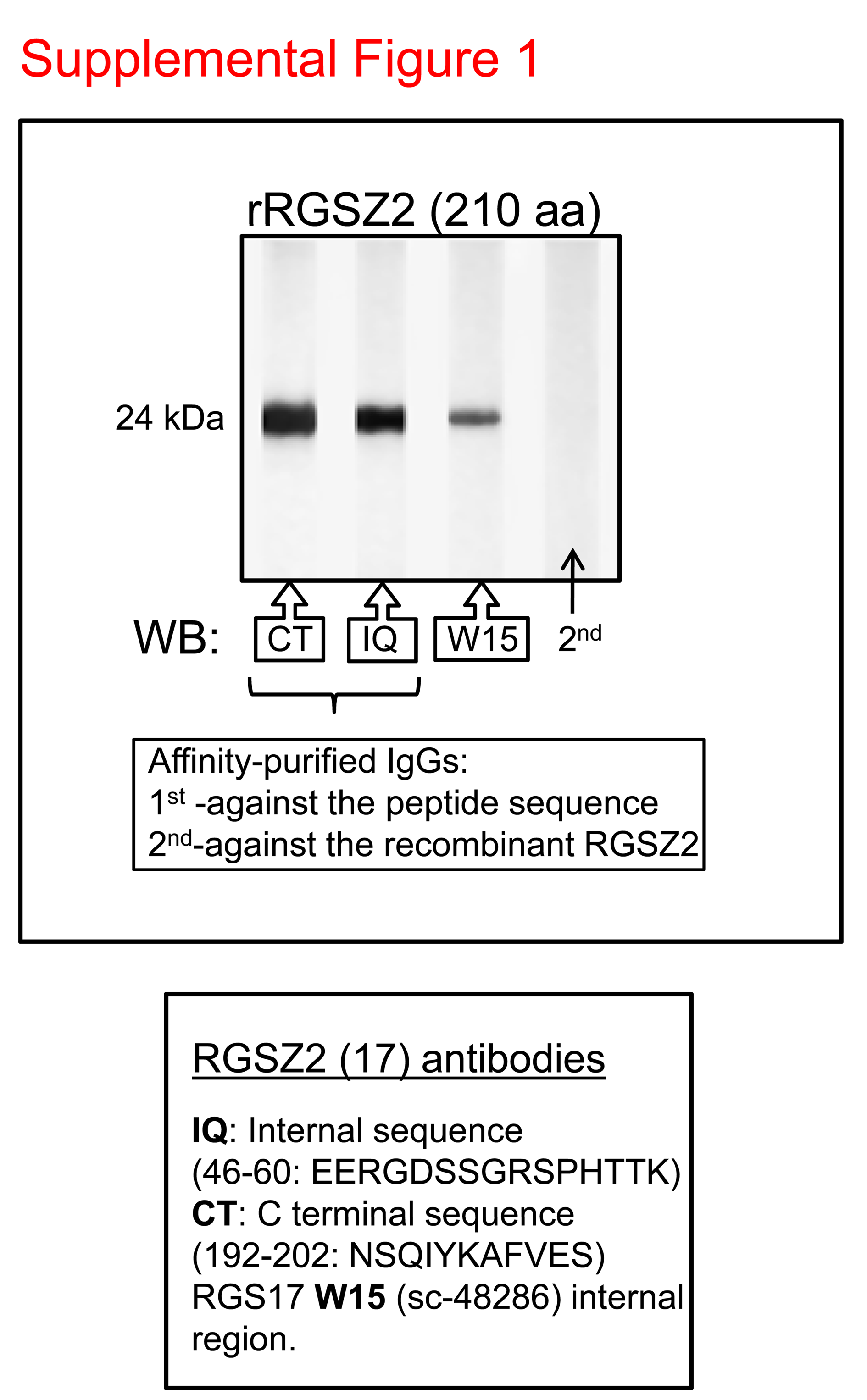

Supplement: Figure S1 — Supporting figure (TIF) [file pone.0028557.s001.tif]
